# Supplementary material for: The Inhibitory Mechanism of 7H-Pyrrolo[2,3-d]pyrimidine Derivatives as Inhibitors of P21-Activated Kinase 4 through Molecular Dynamics Simulation
Source: Molecules. 2023 Jan 3;28(1):413. doi: 10.3390/molecules28010413 (PMC9823812; doi:10.3390/molecules28010413)
Supplement: Supplementary file 1 [file molecules-28-00413-s001.zip › supporting information.pdf]

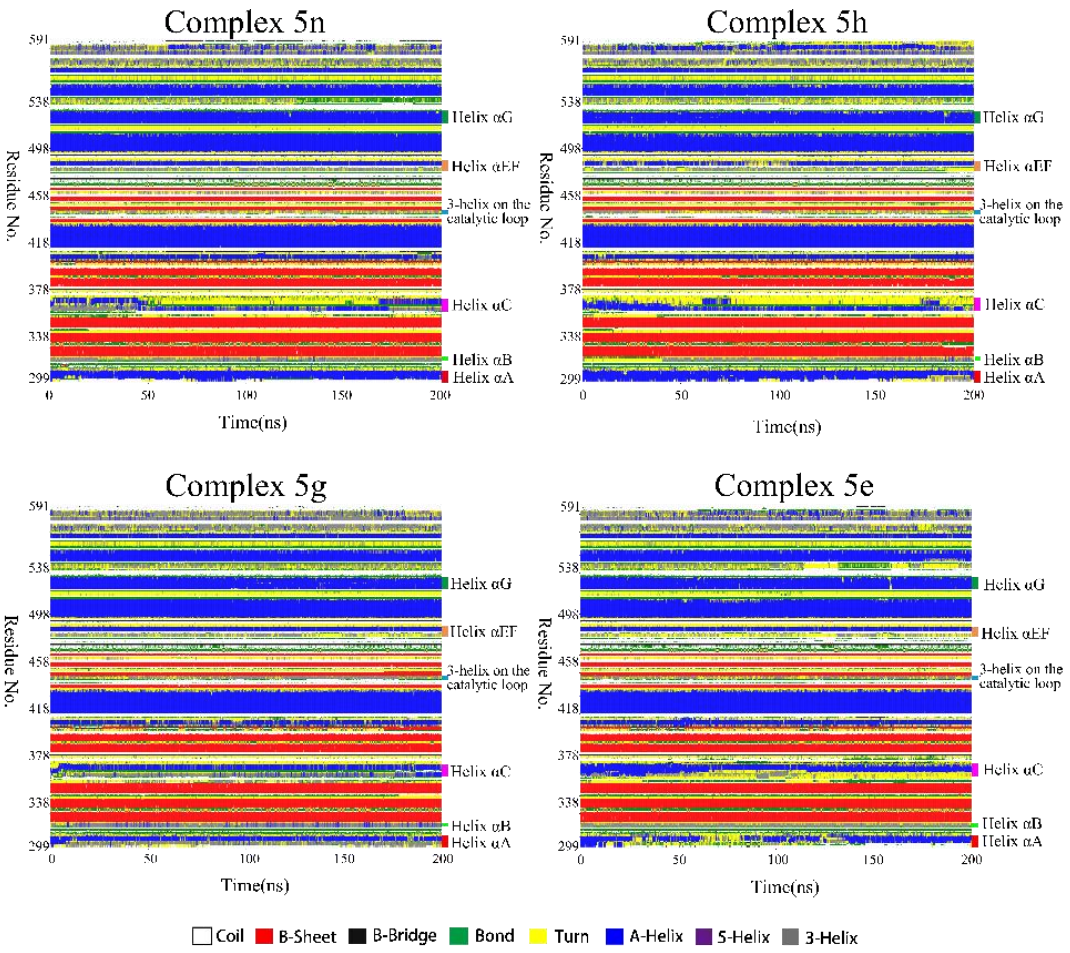

Figure S1. The changes in the secondary structure of proteins in the four complexes with simulation time.

Table S1. The hydrogen bond occupancy rates and the average numbers of hydrogen bonds in the four complexes during the simulation

Complex	Bonding residue	Occurrence	Average number
5n	Leu398	148.29%	2.309
	Glu396	55.83%	
	Ile327	14.43%	
	Asp444	6.35%	
	Glu29	2.95%	
	Gly401	1.12%	

5h	Leu398	152.54%	2.092
	Glu396	52.31%	
	Ile327	1.51%	
	Gly401	0.93%	
5g	Leu398	131.49%	2.031
	Glu396	64.74%	
	Ile327	2.52%	
	Glu329	1.21%	
5e	Leu398	142.04%	2.143
	Glu396	60.96%	
	Glu329	7.13%	
	Ile327	0.65%	

Table S2. Relative binding free energies calculated by MM/PBSA for the four complexes (kJ·mol^{-1})

Energy term	PAK4/5n	PAK4/5h	PAK4/5g	PAK4/5e
ΔG_{vdW}	0	2.374	22.842	24.083
ΔG_{elec}	0	9.037	6.329	16.591
ΔG_{PB}	0	2.175	-11.221	-7.899
ΔG_{SA}	0	0.166	1.766	2.371
ΔG_{polar} [a]	0	11.212	-4.892	8.692
$\Delta G_{nonpolar}$ [b]	0	2.54	24.608	26.454
ΔG_{bind} [c]	0	13.752	19.803	35.162

Table S3. The protonation states of the charged residues

Residues	Lys	Glu	Asp	Arg
Protonation states	protonated	nonprotonated	nonprotonated	protonated
